# Association of Human Papillomavirus 16 E2 with Rad50-Interacting Protein 1 Enhances Viral DNA Replication

**DOI:** 10.1128/JVI.02305-16

**Published:** 2017-02-14

**Authors:** Karen Campos-León, Kalpanee Wijendra, Abida Siddiqa, Ieisha Pentland, Katherine M. Feeney, Alison Knapman, Rachel Davies, Elliot J. Androphy, Joanna L. Parish

**Affiliations:** a University of Birmingham, Institute of Cancer and Genomic Sciences, Birmingham, United Kingdom; b University of St Andrews, School of Medicine, St Andrews, United Kingdom; c Indiana University School of Medicine, Department of Dermatology, Indianapolis, Indiana, USA; University of California, Irvine

**Keywords:** E2, Rad50-interacting protein, papillomavirus, replication

## Abstract

Rad50-interacting protein 1 (Rint1) associates with the DNA damage response protein Rad50 during the transition from the S phase to the G_2_/M phase and functions in radiation-induced G_2_ checkpoint control. It has also been demonstrated that Rint1 is essential in vesicle trafficking from the Golgi apparatus to the endoplasmic reticulum (ER) through an interaction with Zeste-White 10 (ZW10). We have isolated a novel interaction between Rint1 and the human papillomavirus 16 (HPV16) transcription and replication factor E2. E2 binds to Rint1 within its ZW10 interaction domain, and we show that in the absence of E2, Rint1 is localized to the ER and associates with ZW10. E2 expression results in a disruption of the Rint1-ZW10 interaction and an accumulation of nuclear Rint1, coincident with a significant reduction in vesicle movement from the ER to the Golgi apparatus. Interestingly, nuclear Rint1 and members of the Mre11/Rad50/Nbs1 (MRN) complex were found in distinct E2 nuclear foci, which peaked during mid-S phase, indicating that the recruitment of Rint1 to E2 foci within the nucleus may also result in the recruitment of this DNA damage-sensing protein complex. We show that exogenous Rint1 expression enhances E2-dependent virus replication. Conversely, the overexpression of a truncated Rint1 protein that retains the E2 binding domain but not the Rad50 binding domain acts as a dominant negative inhibitor of E2-dependent HPV replication. Put together, these experiments demonstrate that the interaction between Rint1 and E2 has an important function in HPV replication.

**IMPORTANCE** HPV infections are an important driver of many epithelial cancers, including those within the anogenital and oropharyngeal tracts. The HPV life cycle is tightly regulated and intimately linked to the differentiation of the epithelial cells that it infects. HPV replication factories formed in the nucleus are locations where viral DNA is copied to support virus persistence and amplification of infection. The recruitment of specific cellular protein complexes to these factories aids efficient and controlled viral replication. We have identified a novel HPV-host interaction that functions in the cellular response to DNA damage and cell cycle control. We show that the HPV E2 protein targets Rad50-interacting protein 1 (Rint1) to facilitate virus genome replication. These findings add to our understanding of how HPV replicates and the host cell pathways that are targeted by HPV to support virus replication. Understanding these pathways will allow further research into novel inhibitors of HPV genome replication.

## INTRODUCTION

The human papillomavirus (HPV) life cycle is initiated upon infection of undifferentiated keratinocytes localized to the basal layer of the stratified epithelium. Once the 8-kb double-stranded DNA viral genome has entered the nuclear compartment, the viral early proteins E1 and E2 are expressed and recruited to the viral origin of replication (Ori) to initiate the replication of episomal viral genomes and increase copy numbers (1–4). This initial and transient amplification is poorly understood but precedes a stage in which HPV genomes are replicated for stable maintenance and segregation following host cell division (reviewed in reference 5). The expression of the viral E6 and E7 oncoproteins inhibits cell growth arrest and induces proliferation in suprabasal keratinocytes (reviewed in reference 6). In the upper layers of the epithelium, E6 and E7 expression levels are attenuated, resulting in host cell differentiation. This facilitates late promoter activation and the expression of E1∧E4, resulting in the amplification of replication, and the virus capsid proteins L1 and L2, required for the packaging of viral genomes. All of these life cycle events are coordinated through numerous virus-host interactions whereby HPV manipulates important cellular networks to support the persistence of infection and efficient life cycle completion.

Manipulation of the cellular DNA damage response (DDR) by HPV was reported previously. The Mre11-Rad50-Nbs1 (MRN) triprotein complex plays a fundamental role in sensing double-strand DNA breaks (DSBs) and triggers checkpoint activation, which is necessary to maintain the integrity of the cellular genome (7, 8). In the absence of damage, MRN complex proteins are homogeneously localized in the cell nucleus but are recruited to sites of DNA damage, resulting in the formation of large nuclear foci (8). The recruitment of the MRN complex to sites of damage initiates the ataxia telangiectasia mutated (ATM) signaling pathway to activate the G_2_/M checkpoint following DNA damage (9). This occurs as the MRN complex binds to and activates ATM, which in turn phosphorylates several effector proteins, including checkpoint protein 2 (Chk2) and p53 (10). It has also been suggested that MRN participates in the ataxia telangiectasia-related (ATR)-dependent DDR signaling cascade (11).

HPV replication activates the ATM signaling cascade in differentiated keratinocytes, which is required for viral genome amplification. MRN components localize to nuclear replication foci in both undifferentiated and differentiated keratinocytes (12). This phenomenon is not unique to HPV; other small DNA viruses target the MRN complex by altering MRN protein levels, subcellular localization, or both (reviewed in reference 13). For instance, adenovirus 5 (Ad5) infection inhibits the function of the MRN complex via degradation by the E1b55K/E4orf6 viral protein (9). In addition, the Ad5 E4orf3 protein alters the subcellular localization of the MRN complex; both Mre11 and Nbs1 have been identified at foci adjacent to Ad5 replication centers (14). HPV replication centers also recruit other DDR proteins such as phosphorylated histone 2A (γ-H2AX), ATM, Chk2, and Rad51 (12, 15–17). It has been suggested that HPVs target these DDR proteins to allow the formation of viral replication factories and facilitate HPV DNA amplification (16). Indeed, Nbs1 has been shown to contribute to productive HPV replication not only as a sensor but also as an effector protein during the ATM signaling cascade (18).

The specific mechanisms utilized by HPV to manipulate the cellular DDR to facilitate viral DNA replication during distinct life cycle stages remain largely unknown. Recent studies indicate that multiple HPV proteins have the ability to alter the cellular DDR. HPV E7 plays a key role during the formation of HPV replication factories due to its ability to bind to ATM and induce the activation of this kinase and its major substrate, Chk2 (12). In addition, the E7 protein interacts with Rad50 and Nbs1 and increases the levels of these proteins (18). The HPV E1 and E2 proteins cooperate during the initial stages of viral DNA replication, and their interaction is essential for virus replication (19), which is unaffected by the activation of the DDR (20, 21). Furthermore, low- and high-risk HPV E1 proteins promote arrest at the G_1_/S boundary and trigger the activation of the ATM/Chk2 pathway by inducing DSBs in cellular DNA (21, 22).

In this study, we show that HPV16 E2 associates with Rad50-interacting protein 1 (Rint1). Rint1 binds to Rad50 during the late S, G_2_/M, and M phases of the cell cycle and functions in G_2_/M checkpoint activation following DNA damage (23). In addition, Rint1 can inhibit telomerase-independent telomere lengthening by mediating the interaction between Rad50 and the pocket protein family member p130 (24). Rint1 expression is necessary during early mouse development, and it has been demonstrated that Rint1 has tumor suppressor activities and is essential for the maintenance of centrosome integrity and chromosomal stability (25, 26). Indeed, *RINT1* mutations have been shown to present an increased risk of breast and Lynch syndrome spectrum cancers (27), although a larger case-controlled study did not support this finding (28). Conversely, *RINT1* has been shown to be overamplified in some cancers and capable of inducing cellular transformation when inappropriately overexpressed, indicating that Rint1 also has oncogenic properties (29).

Intriguingly, Rint1 function is also important in subcellular vesicle trafficking. The interaction and cross talk between checkpoint control and vesicular trafficking proteins were previously suggested in studies on the effect of the organization of the Golgi apparatus on cellular mitotic entry (30). Both Rint1 and its interacting partner Zeste-White 10 (ZW10) participate during checkpoint control as well as subcellular trafficking (23, 31–33). Studies have shown that Rint1 is localized mainly to the endoplasmic reticulum (ER) (25, 31) and is necessary for membrane trafficking between the ER and the Golgi apparatus by modulating the recruitment of ZW10 to the syntaxin 18 complex (31). Rint1 also participates in endosome-to-*trans*-Golgi network (TGN) trafficking by interacting with TGN soluble *N*-ethylmaleimide sensitive fusion protein (NSF) attachment receptors (SNAREs) (34). To determine the function of the interaction between HPV16 E2 and Rint1 in the HPV life cycle, we have characterized this interaction and provided evidence that Rint1 participates in E2-dependent viral DNA replication.

## RESULTS

### HPV16 E2 interacts with Rint1.

**FIG 1:**
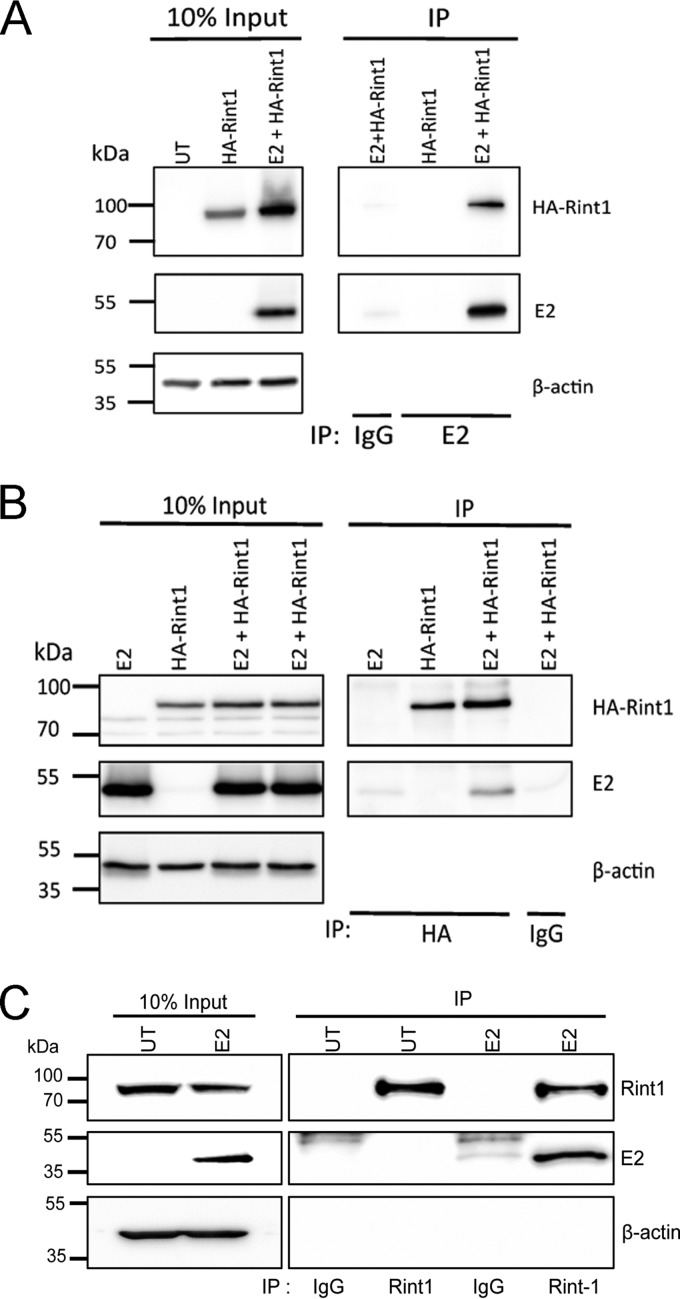
Coimmunoprecipitation of HPV16 E2 and Rint1. (A and B) C33a cells transfected with HA-Rint1 alone or in combination with an HPV16 E2 expression plasmid were lysed and immunoprecipitated with an HPV16 E2-specific antibody (A) or an HA-specific antibody (B). Total and coimmunoprecipitated proteins were detected with HA- and HPV16 E2-specific antibodies. IgG is a nonspecific isotype-matched negative-control antibody used for each immunoprecipitation reaction. (C) C33a cells were left untransfected (UT) or transfected with an HPV16 E2 expression plasmid, and lysates were immunoprecipitated with a Rint1-specific antibody or nonspecific IgG. Total and immunoprecipitated proteins were detected by using Rint1- or HPV16 E2-specific antibodies. The 10% input for each immunoprecipitation is shown on the left. All images shown are representative of results from at least three independent experiments.

During experiments designed to identify novel cellular binding partners of the DNA helicase ChlR1 by a yeast 2-hybrid screen, Rint1 was isolated as a potential candidate (J. L. Parish and E. J. Androphy, unpublished data). Although attempts to verify this interaction by coimmunoprecipitation (co-IP) of exogenous proteins in human cells were unfruitful due to an apparent instability of Rint1 in the presence of overexpressed ChlR1, we noticed that the coexpression of bovine papillomavirus 1 (BPV1) E2, which targets ChlR1 for the stable maintenance of BPV1 genomes (35, 36), was able to stabilize the Rint1 protein in the presence of exogenously expressed ChlR1. We therefore hypothesized that Rint1 may also be targeted by, and form a complex with, the papillomavirus E2 protein. To test this, lysates of C33a cells expressing HPV16 E2 and the hemagglutinin (HA)-tagged Rint1 protein were immunoprecipitated with HPV16 E2-specific antibodies, and coimmunoprecipitation of HA-Rint1 was determined by Western blotting (Fig. 1A). HA-Rint1 was efficiently coimmunoprecipitated in lysates expressing HPV16 E2 but not in the absence of E2 expression. Immunoprecipitation with nonspecific rabbit IgG did not precipitate HA-Rint1, demonstrating the specificity of the assay. The reverse coimmunoprecipitation was also possible; immunoprecipitation with HA-specific antibodies resulted in the coimmunoprecipitation of E2 when the two proteins were coexpressed but not when E2 was expressed alone (Fig. 1B). Coimmunoprecipitation of HA-Rint1 with BPV1 E2 was also possible, demonstrating that this interaction is likely to be conserved among diverse papillomavirus types (data not shown). Furthermore, we were able to coimmunoprecipitate the HPV16 E2 protein with endogenous Rint1 (Fig. 1C), indicating that this interaction occurs at physiological levels of Rint1 and that the HA epitope tag fused to exogenously expressed Rint1 does not induce binding.

**FIG 2:**
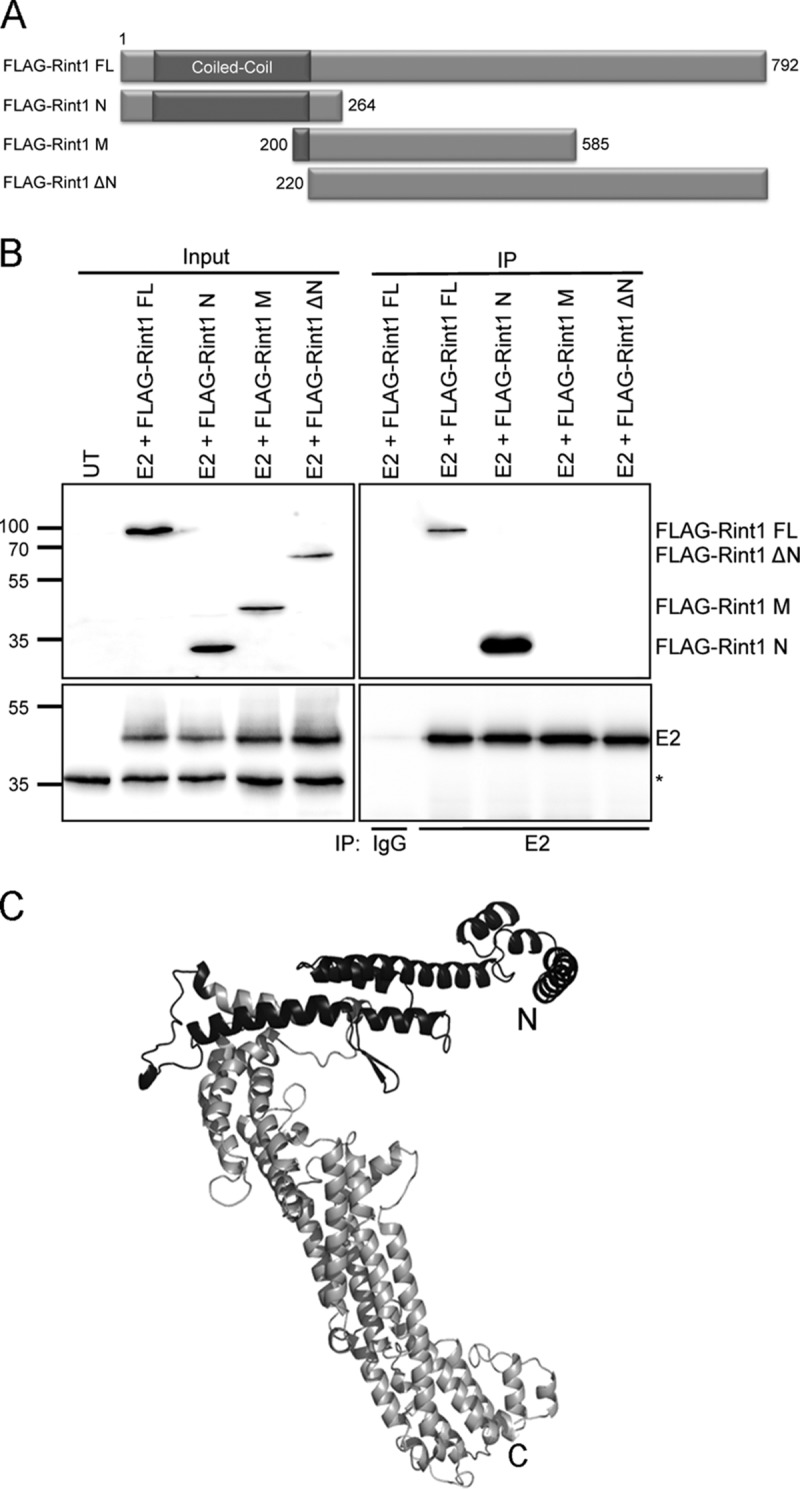
Domain mapping of the E2 interaction site within Rint1. (A) Schematic representation of full-length (FL) Rint1 and truncations used for domain mapping experiments. N, amino acids 1 to 264; M, amino acids 200 to 585; ΔN, amino acids 220 to 792. (B) C33a cells were transfected with HPV16 E2 and FLAG-Rint1 expression plasmids (full-length or truncated Rint1 proteins). Cell lysates were immunoprecipitated with HPV16 E2-specific antibody (sheep) or nonspecific IgG. Total and immunoprecipitated proteins were detected by using FLAG- or HPV16 E2-specific antibodies (TVG261). The input of total cell lysates is shown on the left, which also shows a nonspecific cross-reacting band to indicate loading (*). The data shown above are representative of results from four independent experiments. (C) Molecular model of amino acids 70 to 783 of Rint1 obtained by using Phyre, modeled on the crystal structure of the Saccharomyces cerevisiae Rint1 homologue Tip20 (53). Amino acids 1 to 69 and 784 to 792 were excluded from the model, as they do not have significant homology to Tip20. The image was produced by using PyMOL, and the E2/ZW10 (31) binding region is highlighted in dark gray at the N terminus (N). The Rad50 binding region of Rint1 is contained within the light gray C-terminal (C) region (23).

To map the E2 binding domain within Rint1, we utilized a series of epitope-tagged truncated Rint1 proteins (Fig. 2A) and assayed their binding to E2 by a coimmunoprecipitation assay. In this assay, E2 bound to the full-length FLAG-tagged Rint1 protein, confirming the results shown in Fig. 1. E2 bound to amino acids (aa) 1 to 264 of Rint1 (FLAG–Rint1-N) but did not bind to amino acids 200 to 585 (FLAG–Rint1-M) or amino acids 220 to 792 (FLAG–Rint1-ΔN), indicating that E2 associates with the N-terminal 200 amino acids of Rint1 (Fig. 2B). Interestingly, this binding site is distinct from the Rad50 binding site, which was previously mapped between amino acids 257 and 792 of Rint1 (23) but overlaps the ZW10 binding site (shown to bind to amino acids 1 to 264) (31), suggesting that E2 may disrupt the association of Rint1 with ZW10 but not Rad50 (Fig. 2C).

### HPV16 E2 relocalizes Rint1 to the nucleus and forms Rint1-associated nuclear foci.

**FIG 3:**
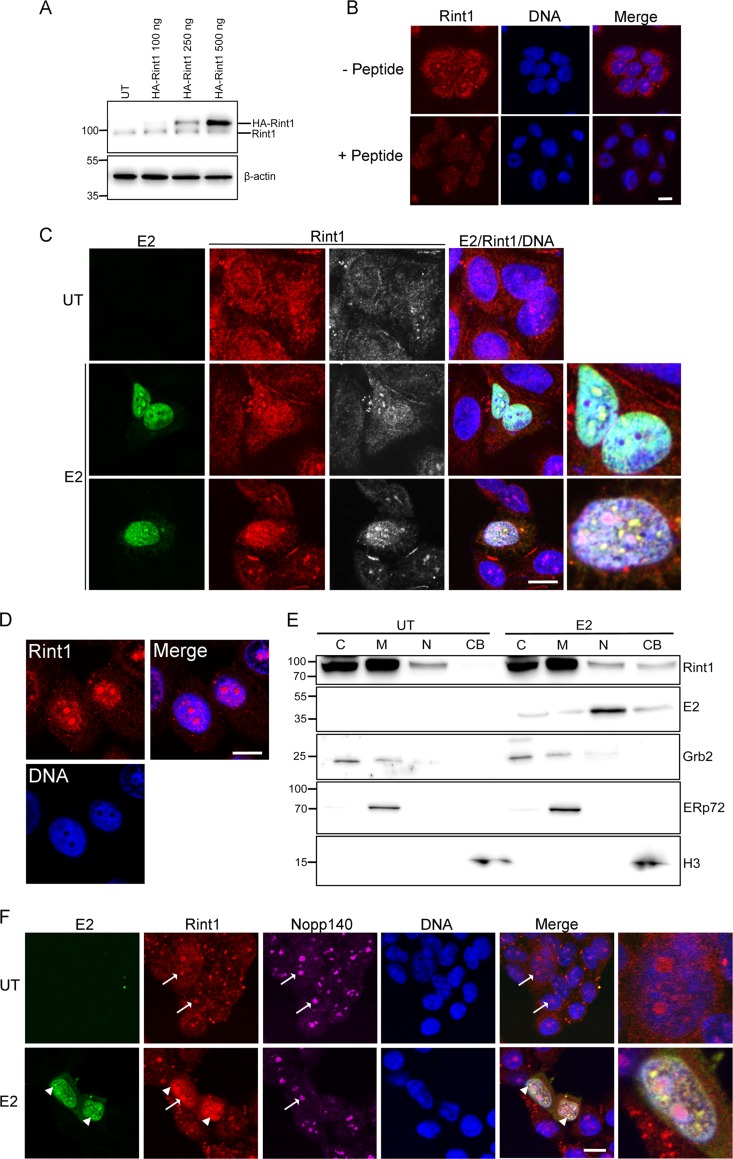
HPV16 E2 expression causes nuclear accumulation of Rint1. (A and B) Validation of the Rint1 antibody used for detection of endogenous Rint1 was performed by Western blot analysis of untransfected (UT) C33a cell lysates and lysates from cells transfected with increasing amounts of an HA-Rint1-expressing plasmid (A) and IF detection in the absence (−) or presence (+) of a Rint1 antibody blocking peptide (B). (C) Localization of E2 and endogenous Rint1 was determined in C33a cells that were either untransfected or transfected with an HPV16 E2 expression plasmid. Cells were fixed and stained with a Rint1-specific antibody (red/gray), E2 TVG261 antibody (green), and the appropriate Alexa Fluor-conjugated secondary antibodies. Enlarged images are shown on the right. Colocalization was quantified by using ImageJ to calculate Manders' overlap coefficient; 95.4% of the E2 protein colocalized with Rint1 (SD, ±5.3%; *n* = 56). (D) Rint1 localization (red) and DNA staining (blue) in HPV16 episome-containing W12E cells. (E) Nuclear accumulation of the Rint1 protein in untransfected and E2-expressing cells was determined by subcellular fractionation followed by Western blotting of Rint1 and E2 proteins alongside markers for the cytoplasmic (C) (Grb2), membrane (M) (ERp72), and chromatin-bound (CB) (histone H3) fractions. (F) Untransfected or E2-transfected cells were costained with E2 (green)-, Rint1 (red)-, and Nopp140 (magenta)-specific antibodies to observe nucleolar localization. The white arrows indicate Nopp140-positive nucleoli that also contain the Rint1 protein, and the white arrowheads indicate E2/Rint1-positive nuclear foci that do not costain with Nopp140. Enlarged images are shown on the right. Bars, 10 μm.

To determine the biological function of the interaction between Rint1 and E2, we next analyzed the localizations of both proteins by immunofluorescence (IF) analysis. For these experiments, we used a commercially available antibody validated by Western blotting of cell lysates of untransfected cells to detect endogenous Rint1 and lysates of cells transfected with increasing amounts of an HA-Rint1 protein expression plasmid (Fig. 3A). Immunofluorescent staining of methanol-fixed cells revealed that the subcellular localization of endogenous Rint1 in C33a cells was predominantly perinuclear, with some large, prominent nuclear foci being visible (31) (Fig. 3B). To further demonstrate the specificity of the antibody, peptide blocking experiments were performed, in which the majority of the fluorescent signal was absent following incubation of the antibody with a specific blocking peptide (Fig. 3B).

The localization pattern of Rint1 was altered in cells expressing HPV16 E2, where an increase in nuclear Rint1 staining that diffusely colocalized with E2 was observed. Furthermore, in a subset of cells, Rint1 colocalized with E2 in nuclear foci, which were brighter, smaller, and present in larger numbers than the nuclear foci observed in the absence of E2 (Fig. 3C). Analysis of colocalization in E2-expressing cells by using Manders' overlap coefficient revealed that 95.4% (standard deviation [SD], ±5.3%; *n* = 56) of the E2 protein colocalized with endogenous Rint1. To determine whether the relocalization of Rint1 to the nuclear compartment is physiologically relevant, the Rint1 localization in HPV16 genome-containing W12E cells was determined. These cells maintain ∼100 to 200 HPV16 episomes per cell (37) (data not shown). The Rint1 protein was clearly present in the nuclear compartment of these cells (Fig. 3D), indicating that E2 expressed at physiological levels from the viral genome is able to relocate a pool of Rint1 to the nucleus of infected cells. This apparent relocalization of Rint1 by E2 was corroborated by subcellular fractionation experiments in which an increase in the amount of chromatin-bound Rint1 was reproducibly observed in E2-transfected cells compared to untransfected control cells (Fig. 3E).

To further characterize E2-Rint1 nuclear foci, and to determine whether these foci were distinct from the larger Rint1 foci observed in untransfected cells (Fig. 3B), untransfected or E2-transfected cells were costained with E2, Rint1, and Nopp140 antibodies (Fig. 3F). Nopp140 is a nucleolus-specific protein previously used as a marker of nucleolar localization (38). In the absence of E2, Rint1 colocalized with Nopp140-positive foci in the majority of cells, providing evidence that Rint1 associates with nucleoli. These Rint1-Nopp140-positive foci were also present in E2-expressing cells; however, smaller Rint1 foci that were E2 positive but Nopp140 negative were also formed (Fig. 3F). These data provide evidence that E2 relocalizes Rint1 to nuclear foci that are not associated with nucleoli.

### HPV16 E2 disrupts the Rint1-ZW10 interaction.

**FIG 4:**
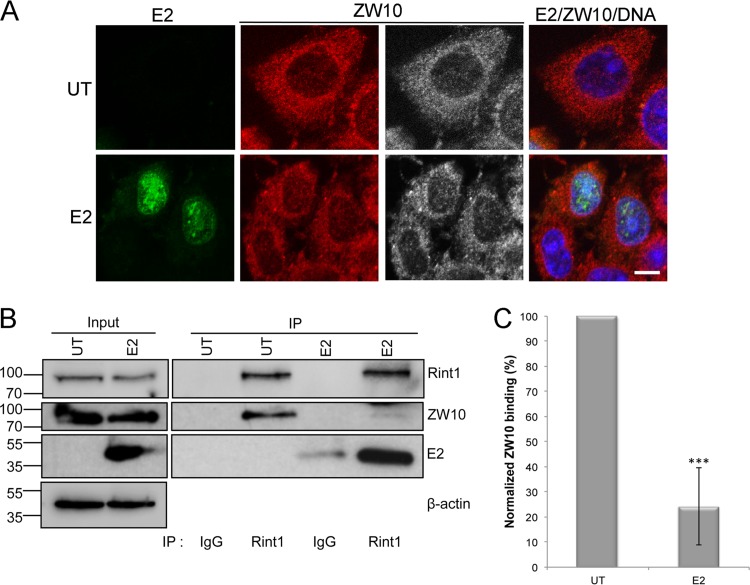
HPV16 E2 disrupts the Rint1-ZW10 interaction. (A) E2-transfected cells were stained with E2 TVG261 (green), ZW10-specific antibodies (red/gray), and the appropriate Alexa Fluor-conjugated secondary antibodies. In both panels, DNA was stained with Hoechst 33342 (blue). Bar, 5 μm. (B) C33a cells were untransfected (UT) or transfected with an E2-expressing plasmid, and lysates were immunoprecipitated with Rint1-specific antibodies or an IgG isotype-matched control. Immunoprecipitates were divided equally and loaded onto 2 separate gels to simultaneously detect Rint1 and ZW10, which have similar molecular weights. Rint1, ZW10, E2, and β-actin were detected by Western blotting in the input (left) and immunoprecipitates (right), as indicated. (C) The percentage of ZW10 that coimmunoprecipitated with Rint1 in the absence (untransfected; UT) or presence (E2) of E2 protein expression was quantified by densitometry using ImageJ. The data shown are means and standard deviations from 4 independent experiments (***, *P* < 0.001).

An essential role for Rint1 in subcellular trafficking, through its interaction with ZW10, has been well documented (31, 34, 39). To determine whether ZW10 was also maintained in the nucleus by E2, cells were stained with a ZW10-specific antibody (Fig. 4A). Interestingly, ZW10 was not relocalized to the nuclear compartment in E2-expressing cells as was observed with Rint1, suggesting that E2 disrupts the interaction of Rint1 with ZW10 and results in the accumulation of the Rint1 protein in the nucleus. To confirm that this is the case, Rint1 and ZW10 protein complexes were analyzed by coimmunoprecipitation in the presence and absence of E2 protein expression (Fig. 4B). In untransfected cells that do not express E2, robust coimmunoprecipitation of endogenous ZW10 with Rint1 antibody was observed. In agreement with our hypothesis, E2 expression in the same cell line reduced the proportion of ZW10 associated with Rint1 but did not have any effect on the expression level of either Rint1 or ZW10. Data from three independent repetitions of this experiment were quantified by using ImageJ, demonstrating a 75.7% ± 15.4% (SD) (*P* < 0.001) reduction in the amount of ZW10 bound to Rint1 in the presence of E2. These experiments show that E2 efficiently disrupts the Rint1-ZW10 interaction, suggesting that E2 targets the pool of Rint1 protein that is in complex with ZW10 and functions in subcellular trafficking.

**FIG 5:**
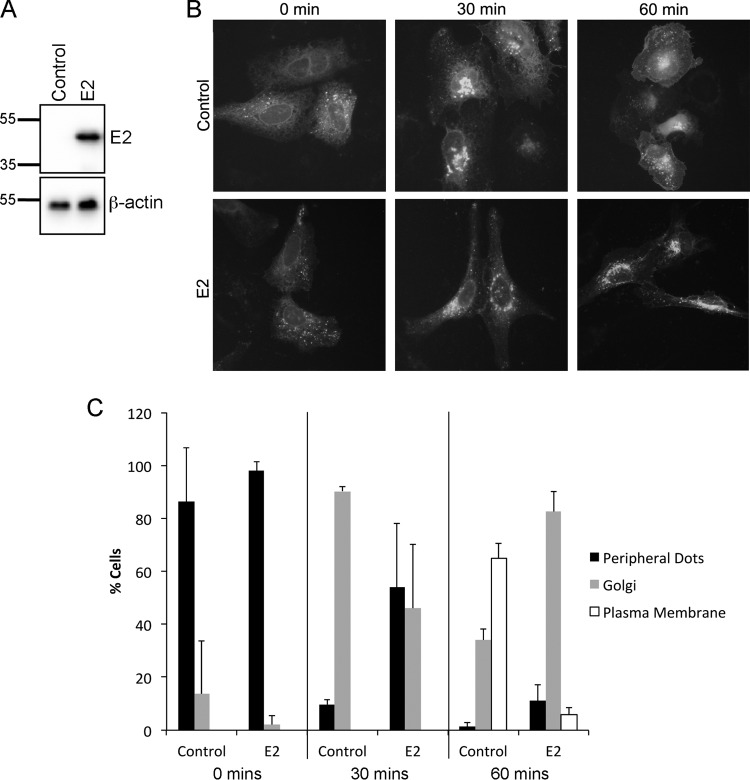
HPV16 E2 causes delayed EGFP-VSVG transport from the ER. U2OS cells stably expressing HPV16 E2 or the equivalent empty vector control were used to study EGFP-VSVG trafficking. (A) E2 expression was determined by Western blotting using an HPV16 E2-specific antibody. Membranes were subsequently stripped, and β-actin was detected as a loading control. (B) E2-expressing or control cells were transfected with the EGFP-VSVG-expressing plasmid, and cells were incubated at 40°C for 24 h. Cells were then transferred to 32°C to allow vesicle transport, which was visualized at 0, 30, and 60 min. (C) Quantification of VSVG-EGFP localization at each time point. A total of 200 cells were scored for each time point. The data show the means and standard errors from three independent experiments.

Given that E2 expression disrupts the Rint1-ZW10 interaction and results in the accumulation of at least a proportion of endogenous Rint1 protein but not ZW10 in the nuclear compartment (Fig. 3C and 4) and that Rint1 is essential for vesicle trafficking through its interaction with ZW10 (31), we hypothesized that this would result in an attenuation of vesicle movement in E2-expressing cells. To test this, U2OS cells that stably express the HPV16 E2 protein or the empty vector control (Fig. 5A) (40) were transfected with a pEGFP-VSVG plasmid, which expresses a temperature-sensitive mutant vesicular stomatitis virus-encoded glycoprotein (VSVG) fused to enhanced green fluorescent protein (EGFP) (41). When cells are incubated at 40°C, EGFP-VSVG is unable to exit the ER. Shifting the cells to 32°C allows movement from the ER to the Golgi apparatus and finally to the plasma membrane. In control cells, EGFP-VSVG had moved to the Golgi apparatus at 30 min, whereas vesicle movement in E2-expressing cells was significantly delayed (*P* < 0.05). This effect was even more dramatic at 60 min; whereas EGFP-VSVG in control cells had reached the plasma membrane in over 60% of cells, only a small number of E2-expressing cells had EGFP-VSVG at the plasma membrane (*P* < 0.001). The majority of these cells still retained EGFP-VSVG at the Golgi apparatus (Fig. 5B and C).

### HPV16 E2 and Rint1 colocalize throughout the cell cycle and are recruited to nuclear foci during S phase.

**FIG 6:**
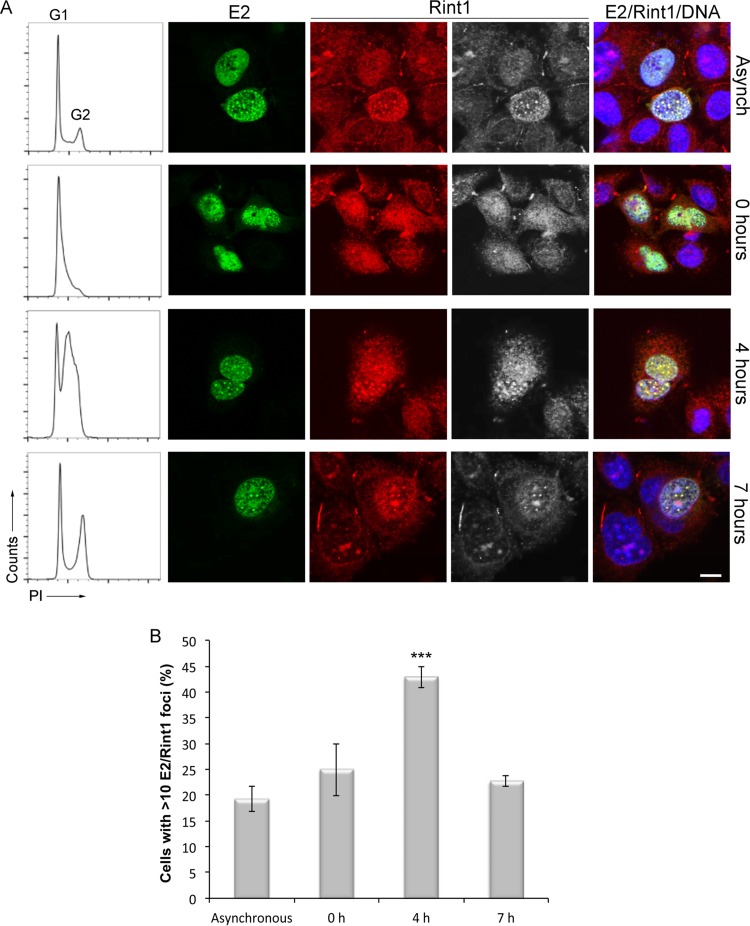
HPV16 E2 and Rint1 colocalize in nuclear foci during S phase. C33a cells were transfected with an HPV16 E2-expressing plasmid and synchronized by a double-thymidine block. Asynchronously growing cells or cells harvested 0, 4, or 7 h following release were fixed and stained with propidium iodide (PI), and cell cycle profiles were obtained by flow cytometry (left). Cells within the same cultures were fixed at the indicated time points. Localization of E2 and Rint1 was determined by staining with the E2-specific TVG261 antibody (green) and Rint1 antibody (red/gray). DNA was stained with Hoechst 33342 (blue). Bar, 5 μm. (B) Quantification of E2-expressing cells containing >10 E2/Rint1-positive foci at each time point. A total of 500 cells were scored at each time point. The data shown are the means and standard deviations from three independent experiments (***, *P* < 0.001 compared to asynchronous cells).

Since E2 and Rint1 diffusely colocalized in the nucleus of some E2-transfected cells, while E2 and Rint1 colocalized in distinct nuclear foci in other cells (Fig. 3C), we hypothesized that these two different phenotypes are cell cycle dependent. To test this, E2-transfected cells were synchronized at the G_1_/S boundary by a double-thymidine block and fixed at 0 h (G_1_/S phase), 4 h (mid-S phase), and 7 h (G_2_/M phase) postrelease. Cell synchronization was confirmed by flow cytometry, and these cells were compared to an asynchronously growing population (Fig. 6A). The subcellular localization of E2 and endogenous Rint1 was then detected by immunofluorescence staining, and the number of cells containing more than 10 E2/Rint1 foci (E2/Rint1^>10^) at each time point was evaluated. In asynchronous cultures, and in accordance with our above-described observations, the majority of cells had E2 and Rint1 distributed homogeneously within the nucleus, and 19.2% (SD, ±4.2%) of cells had distinct E2/Rint1 nuclear foci. Similarly, E2 and Rint1 were uniformly distributed within the nucleus of the majority of cells synchronized at the G_1_/S boundary, and 25.0% (SD, ±8.7%) of cells contained E2/Rint1^>10^-positive foci. Interestingly, the colocalization of E2 and Rint1 in distinct nuclear foci was most evident in cells harvested at mid-S phase, where 42.9% (SD, ±3.6%) of cells had E2/Rint1^>10^-positive foci. The proportion of cells with E2/Rint1^>10^-positive foci peaked in mid-S phase and was reduced to 22.7% (SD, ±1.9%) as cells entered G_2_/M phase. In addition to the reduction in the number of cells with >10 E2/Rint1 foci at the G_2_/M phase, the foci present in these cells were generally smaller than those that had been observed during the mid-S phase.

Overall, these data demonstrate that HPV16 E2 and endogenous Rint1 colocalize throughout the cell cycle. Moreover, E2 and endogenous Rint1 are recruited to distinct nuclear foci, and the formation of these structures is enhanced during S phase.

### HPV16 E2 partially colocalizes with MRN complex proteins Mre11 and Nbs1.

**FIG 7:**
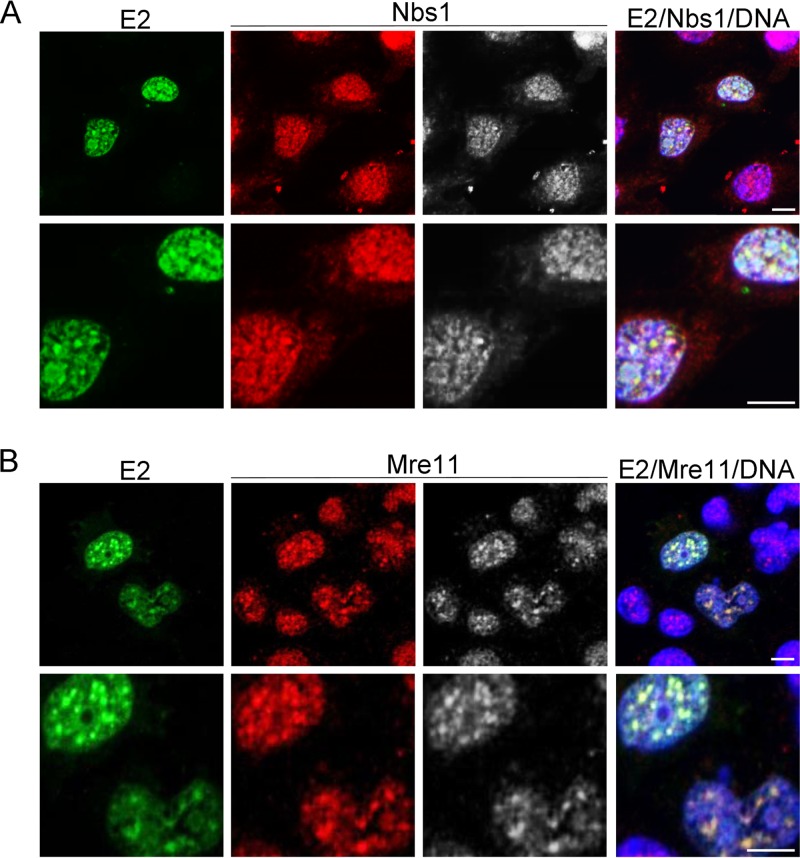
HPV16 E2 partially colocalizes with the MRN complex proteins Mre11 and Nbs1. C33a cells were transfected with an HPV16 E2 expression plasmid, fixed in ice-cold methanol, and stained with an E2-specific antibody (green). DNA was stained with Hoechst 33342 (blue). (A) Endogenous Nbs1 was detected with an Nbs1-specific antibody (red/gray). (B) Endogenous Mre11 was detected with an Mre11-specific rabbit antibody (red/gray). Bar, 5 μm. Digital zoom is shown at the bottom.

Previous reports showed that Rint1 interacts with the DNA repair protein Rad50 (23, 24). Since Rad50 forms part of the Mre11/Rad50/Nbs1 (MRN) complex, which is important in DNA damage repair and checkpoint surveillance, we examined whether the members of this complex were also recruited to E2-containing foci. C33a cells were transfected with an HPV16 E2 expression plasmid, and the subcellular localization of E2 as well as endogenous Nbs1, Mre11, and Rad50 was analyzed by immunofluorescence microscopy following fixation of cells in −20°C methanol. Unfortunately, we were not able to detect Rad50 due to poor staining with the Rad50-specific antibody used. Punctate nuclear staining of Nbs1 and Mre11 was observed in untransfected C33a cells, as was previously observed for unperturbed cells by using similar fixation methods (42). Transfection of cells with HPV16 E2, however, resulted in the formation of more strongly stained Nbs1 and Mre11 nuclear foci that partially colocalized with E2 (Fig. 7A and B).

### Rint1 colocalizes with HPV16 E2/E1 foci in the presence of the viral origin of replication.

**FIG 8:**
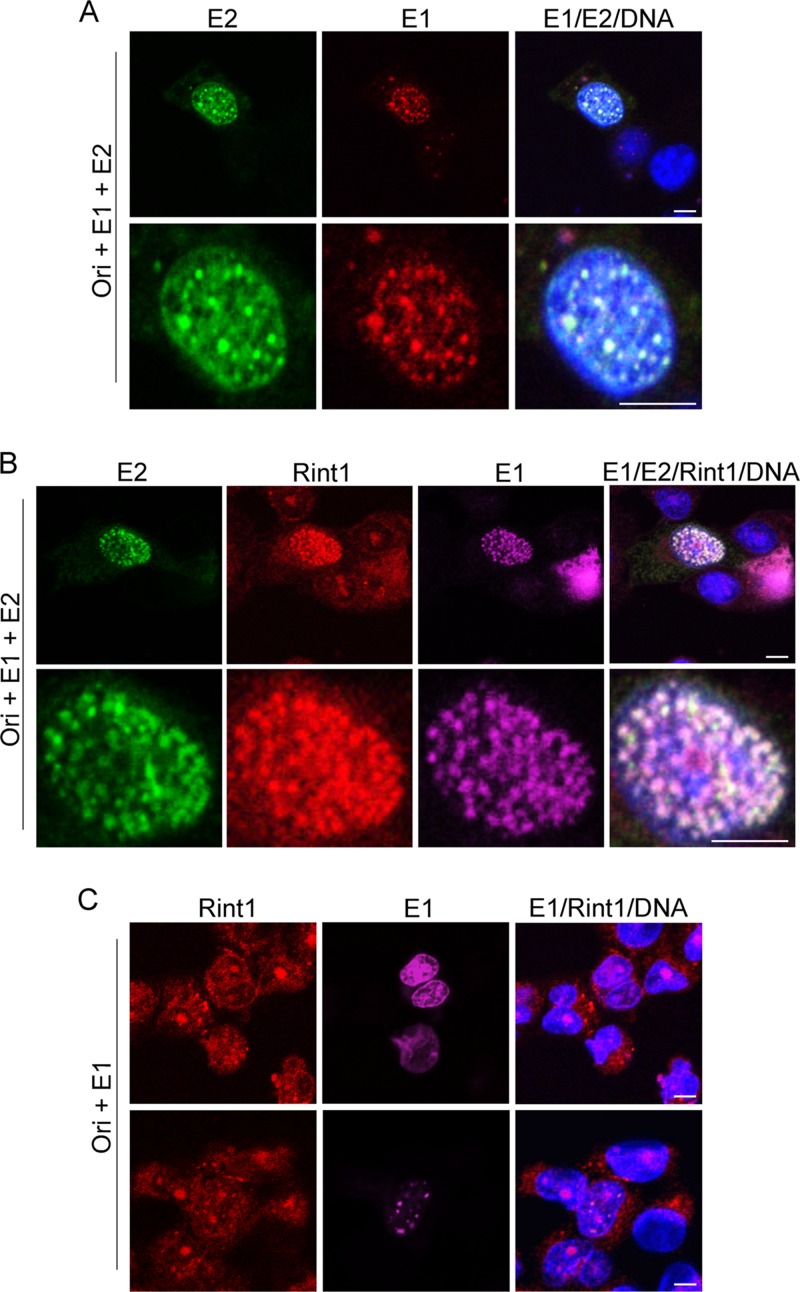
Rint1 colocalization to E1/E2 replication foci is E2 dependent. (A) C33a cells were cotransfected with HPV16 Ori (p16OriM), HPV16 E2, and HPV16 HA-E1 expression plasmids. Cells were fixed, and E2 (green) and HA-tagged E1 (red) were detected with E2- and HA-specific antibodies, respectively. Cellular DNA was stained with Hoechst 33342 (blue). (B) Cells were transfected as described above for panel A, and Rint1 (red) was detected along with E1 (magenta) and E2 (green). (C) Cells were transfected with p16OriM and HPV16 HA-E1 in the absence of E2, and Rint1 (red) and E1 (magenta) protein localization was detected as described above for panel B. Bar, 5 μm. Digital zoom is shown at the bottom (A and B).

The subcellular localization of the HPV16 E1 and E2 proteins was previously examined by using different epithelial cell lines, and the coexpression of these viral proteins brings about the formation of discrete nuclear foci, to which proteins that participate during the DNA damage response have also been shown to be recruited (15, 18, 21, 22, 43). In order to determine whether Rint1 is also present in E2/E1-positive foci, C33a cells were cotransfected with HPV16 E2 and HPV16 HA-E1 expression plasmids as well as with a plasmid that contains the HPV16 origin of replication (pOri16M). The subcellular localization of endogenous Rint1 in HPV16 E2/E1-expressing cells was then determined by immunofluorescence staining. As expected from previous reports, HPV16 E1 and E2 colocalized in nuclear foci in the presence of Ori (Fig. 8A). Interestingly, endogenous Rint1 colocalized with E1/E2 foci in the presence of Ori, indicating that Rint1 may be recruited to nuclear virus replication factories (Fig. 8B).

Our results demonstrate that HPV16 E2 alters the subcellular localization of endogenous Rint1 and brings about its relocalization to the nucleus. To rule out the possibility that E1 contributes to the recruitment of Rint1 to E1/E2 replication factories, cells were cotransfected with HPV16 HA-E1 expression plasmid and p16OriM only. The localization of endogenous Rint1 in E1-expressing cells was then determined. While HPV16 E1 was mainly diffusely localized within the nuclei of transfected cells (Fig. 8C, top), a subset of cells within the same population had clear nuclear E1 foci (Fig. 8C, bottom). However, the expression of HPV16 E1 did not change the subcellular localization of endogenous Rint1, which was predominantly localized to punctate perinuclear ER-associated structures and nucleoli, as observed in untransfected cells (Fig. 3B).

### Rint1 overexpression enhances E2-dependent replication.

**FIG 9:**
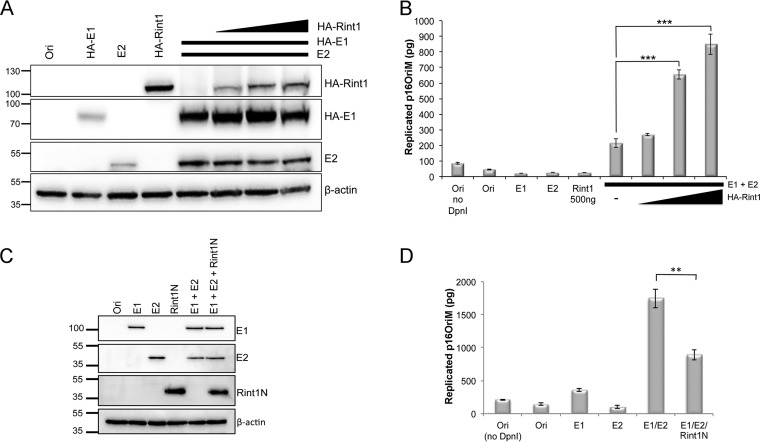
Effect of Rint1 and Rint1-N overexpression on E2-dependent virus replication. C33a cells were transfected with HPV16 Ori (p16OriM), HPV16 E2, and HA-E1 either alone or in combination with increasing amounts of an HA-Rint1 expression plasmid (A and B) or a FLAG–Rint1-N expression plasmid (C and D). (A and C) Cell lysates were analyzed by Western blotting using HPV16 E2-specific, HA-specific (to detect E1 and the full-length Rint1 protein) or FLAG-specific (to detect the FLAG–Rint1-N protein) antibodies. Western blots were stripped and reprobed with a β-actin-specific antibody as a loading control. (B and D) Transient DNA replication of p16OriM was measured by qPCR and compared to a standard curve generated by serial dilution of the purified plasmid. The data shown are means and standard deviations from technical replicates (**, *P* < 0.01; ***, *P* < 0.001). The results are the means and standard errors from a single experiment performed in triplicate and are representative of data from at least three independent repetitions of each experiment.

Our data demonstrate that Rint1 is present in E2/E1-positive foci (Fig. 8B), which were previously shown to be viral replication factories (16); we therefore evaluated the effect of Rint1 overexpression on viral DNA replication. Before performing these experiments, we first determined whether Rint1 expression had any effect on the cell cycle distribution, since an alteration of cell cycle progression would have an indirect effect on virus replication. Cells were transfected with full-length Rint1 and the truncated form, Rint1-N, used in previous experiments, and the cell cycle profile for each sample was determined by flow cytometry. No effects on cell cycle progression were observed following the overexpression of full-length or truncated Rint1 (data not shown). Therefore, cells were transfected with HPV16 E1 and HPV16 E2 either alone or in combination with increasing amounts of HA-tagged Rint1 in the presence of p16OriM. The expression level of each protein was determined by Western blotting (Fig. 9A), and p16OriM replication was determined by quantitative PCR (qPCR) (Fig. 9B). While the replication of p16OriM was minimally detectable in cells transfected with p16OriM alone or cotransfected with either E1, E2, or Rint1 expression plasmids alone, the coexpression of E1 and E2 dramatically enhanced viral origin replication. The coexpression of increasing amounts of HA-Rint1 resulted in a dose-dependent increase in Ori levels, providing evidence that Rint1 stimulates HPV DNA replication (Fig. 9B). In contrast, Rint1 overexpression did not have any significant effect on E2-dependent transcription activation using a synthetic E2-dependent reporter (data not shown).

### Overexpression of the truncated Rint1 protein attenuates E2-dependent virus replication.

Our experiments showed that HPV16 E2 binds to the N-terminal 264 amino acids of Rint1 (Rint1-N) (Fig. 2), a region that was previously shown to be unable to bind to Rad50 (23). Since we observed that the MRN complex proteins Mre11 and Nbs1 were recruited to E2 foci (Fig. 6) and others demonstrated that these proteins are recruited to HPV replication centers (18), we hypothesized that the overexpression of the E2 binding portion of Rint1 may attenuate virus replication. Cells were therefore transfected with viral Ori, E1, and E2 as described above, alone or in combination with a plasmid expressing Rint1-N. Protein expression levels were determined by Western blotting (Fig. 9C), and origin replication was determined by qPCR (Fig. 9D). The overexpression of Rint1-N significantly (*P* < 0.01) inhibited E1/E2-dependent origin replication. Taken together, these results indicate a role for Rint1 in HPV DNA replication.

## DISCUSSION

In this article, we report a novel interaction between the HPV16 E2 protein and the cellular Rad50-binding protein Rint1. Intriguingly, Rint1 seems to have two very different cellular functions. It was originally isolated as a Rad50-binding protein and shown to play a significant role in radiation-induced G_2_ checkpoint activation (23), a function that has been confirmed in the fission yeast homologue damage response protein 1 (Drp1) (44). An essential role for Rint1 in subcellular vesicle trafficking by mediating interactions between ZW10 or Cog1 and members of the SNARE complex at the ER and TGN, respectively, has also been documented (31, 34, 45). In our study, the interaction between HPV16 E2 and Rint1 was mapped to the N-terminal ZW10 binding region of Rint1, and we provide evidence that E2 disrupts the interaction between ZW10 and Rint1, resulting in impaired subcellular vesicle trafficking and the accumulation of Rint1 but not ZW10 in the nucleus. A similar reduction in vesicle trafficking following the overexpression of the truncated Rint1-N protein was described previously (31) and supports our findings that E2 indeed affects Rint1 function in vesicle trafficking. Interestingly, E2 has been shown to associate with other proteins involved in regulating vesicle movement, suggesting that E2 may have multiple effects on subcellular trafficking (46). Why E2 disrupts cellular vesicle movement is not understood. One possible explanation is that disruption is required for efficient virus entry. However, although BPV1 E2 has been shown to be packaged in pseudovirions (47), the presence of E2 in HPV virions has yet to be proven. It is therefore unknown whether E2 plays any role in virus entry through its effect on subcellular vesicle trafficking. An alternative explanation is that E2 disrupts vesicle trafficking to attenuate antigen presentation on the surface of infected cells, thus preventing immune activation. Many other viruses have been shown to inhibit ER-to-Golgi trafficking to prevent major histocompatibility complex (MHC) class I presentation, and it will be interesting to determine whether this is also the case for HPV.

Our data show that endogenous Rint1 colocalizes with E2 nuclear foci, predominantly in the mid-S phase, and that the recruitment of Rint1 to these foci is not dependent on the presence of viral DNA or the E1 viral helicase. While the formation of nuclear foci by the E2 protein expressed in the absence of the HPV origin and the E1 helicase has not been widely reported, our experiments clearly demonstrate that the E2 protein forms nuclear foci in a subset of cells when expressed alone. Similar E2 foci were demonstrated previously, although the function of these foci was not described (46). The E2 foci observed in our experiments were more visible following fixation of cells with ice-cold methanol rather than with paraformaldehyde, which was required to visualize the Rint1 protein with available antibodies. Methanol fixation can remove soluble non-chromatin-bound proteins (48), suggesting that there are E2 foci in the nuclei of some E2-expressing cells but that these foci are less likely to be visible following fixation with paraformaldehyde, which does not remove soluble nuclear proteins.

Although Rint1 is known to bind to Rad50 (23, 24), it is not known whether the pool of Rad50 targeted by Rint1 is associated with the other members of the MRN complex, Mre11 and Nbs1. We were unable to study the recruitment of Rad50 to E2 foci but found that both Mre11 and Nbs1 are recruited to nuclear E2 foci, suggesting that the interaction with Rint1 may mediate the recruitment of the entire MRN complex to E2 foci and that Rad50 is likely to bind Rint1 while existing as part of the MRN complex, although this is yet to be formally proven. Nonetheless, the recruitment of Rint1, Nbs1, and Mre11 to nuclear E2 foci suggests the formation of an E2-dependent prereplication complex containing Rint1 and the associated MRN complex.

It was previously shown that HPV replication foci recruit DNA damage response proteins such as activated ATM, Chk2, and the MRN complex (15, 18), and the amplification of viral DNA in HPV genome-containing cells requires ATM activation (12). The inhibition of this pathway reduces viral episome copy numbers (48). Our experiments show that Rint1 is also recruited to replication factories that contain E1 helicase and presumably the viral origin of replication. These E1/E2/Rint1 foci were generally larger than the E2/Rint1 foci observed, indicating that Rint1 recruitment is initiated by E2 but that virus replication increases the recruitment of Rint1 to replication centers. In support of this model, E1 was unable to relocalize Rint1 to the nucleus in the absence of E2, even in cells that had clear E1 foci. Interestingly, E1 expression alone results in the activation of a DDR, as evidenced by DNA damage marks such as phosphorylated histone variant H2AX (γH2AX) and Chk2 (21, 22). Our data show that Rint1 is not recruited to these sites of E1-induced damage, providing evidence that DNA damage caused by E1 does not engage Rint1 and that E2 brings Rint1 to the replication complex.

In support of the idea that Rint1 is recruited to viral replication factories to regulate virus replication, we show that Rint1 overexpression stimulates E1/E2-dependent virus origin replication in a transient virus DNA replication assay. In addition, we demonstrate that the expression of the truncated Rint1-N protein, which was previously shown to bind to ZW10 and modulate Rint1 function in vesicle trafficking in a dominant negative manner (31) but is unable to bind to the Rad50 protein (23), has a dominant negative effect on HPV DNA replication. We therefore conclude that the suppression of HPV origin replication following the expression of Rint1-N is due to the disruption of the association between full-length Rint1 and the associated MRN complex with E2 but not the disruption of the Rint1-ZW10 interaction, which our experiments show is already abrogated by the expression of the E2 protein (Fig. 4). We confirmed that the expression of full-length and truncated Rint1 proteins did not cause cell cycle arrest or delay, leading us to conclude that Rint1 is recruited to replication foci by E2 to enhance virus replication rather than indirectly affecting replication by an alteration of cell cycle progression. Whether this is important in maintenance replication or the amplification of viral genomes in differentiating keratinocytes is a question that we are currently addressing.

HPV DNA replication induces a cellular DNA damage response that is exploited to recruit DNA replication complexes to enhance viral DNA replication (reviewed in reference 49). We show for the first time that the Rad50-associated protein Rint1 is targeted by E2 and recruited to replication factories. Rint1 enhanced virus replication, and conversely, a dominant negative truncation of Rint1 reduced virus replication. Further analysis of this interaction in the context of the HPV life cycle is important, but given the data reported here, it is tempting to speculate that Rint1 plays an important role in mediating the recruitment of DNA damage response complexes to HPV replication centers to support virus genome amplification.

## MATERIALS AND METHODS

### Cell culture.

C33a cells are HPV-negative human cervical carcinoma-derived keratinocytes grown in Dulbecco's modified Eagle's medium (DMEM) supplemented with 10% fetal bovine serum (FBS). Stable U2OS lines containing either the pCMV4 vector or pCMV4-HPV16-E2 (provided by Iain Morgan, Virginia Commonwealth University), which expresses HPV16 E2, were grown in DMEM supplemented with 10% FBS and 0.7 mg/ml G418 (40).

Cells were synchronized by a double-thymidine block following incubation in 2 mM thymidine for 16 h and subsequently washed with phosphate-buffered saline (PBS) at 37°C. Cells were grown for 8 h in fresh growth medium, followed by a second incubation with 2 mM thymidine for 16 h. Asynchronously growing cells or cells harvested 0, 4, or 7 h following thymidine release were fixed in methanol for IF analysis or fixed in ethanol and stained with propidium iodide for analysis by flow cytometry as previously described (35). W12E cells were obtained from Paul Lambert (University of Wisconsin) and were cultured on γ-irradiated J2-3T3 fibroblasts in F medium as previously described (37).

### Plasmids.

Human Rint1 cDNA was amplified from an oligo(dT)-primed HeLa cDNA library by using the following primers: 5′-CATGCCATGGAGCTGGGTAGTGAGTGTGTCGCT-3′ and 5′-CGGCTCTAGACCAAAGAAACCTTTTTCTGAAAGACA-3′. Products were digested with NcoI and XbaI and ligated into pSP65-HA digested with the same restriction enzymes. pSP65-HA-Rint1 was then digested with BamHI and XbaI, and HA-Rint1 cDNA was ligated into pcDNA3.1 (Promega) to create pcDNA-HA-Rint1, which expresses the Rint1 protein with an N-terminal HA tag from a cytomegalovirus (CMV) promoter. pOri16M (pOriM) contains the HPV16 origin of replication (50), and pCMV-E2 encodes the wild-type HPV16 E2 protein. pHPV16-HAE1 encodes the HA-tagged HPV16 E1 protein (51). These constructs were provided by Iain Morgan, Virginia Commonwealth University. pCMV-FLAG-Rint1-FL (full length) (aa 1 to 792), pCMV-FLAG-Rint1-N (aa 1 to 264), pCMV-FLAG-Rint1-M (aa 200 to 585), and pCMV-FLAG-Rint1-ΔN (aa 565 to 792) encode FLAG-tagged full-length and truncated Rint1 proteins (31) and were provided by Mitsuo Tagaya, Tokyo University of Pharmacy and Life Science, Japan. The plasmid expressing the EGFP-tagged vesicular stomatitis virus G protein (pEGFP-VSVG) was obtained from Addgene (41).

### Antibodies.

Primary antibodies used for IF analysis are as follows: mouse anti-HPV16 E2 (TVG 261, catalogue number ab17185; Abcam), goat anti-Rint1 (N-15, catalogue number SC-19404; Santa Cruz Biotechnology), rabbit anti-Nopp140 (a gift from Thomas Meier, Yeshiva University), rabbit anti-Mre11 (catalogue number 4895-S; Cell Signaling Technology), rabbit anti-HA (catalogue number ab9110; Abcam), rabbit anti-Nbs1 (catalogue number NB100-143; Novus Biologicals), and rabbit anti-ZW10 (catalogue number ab21582; Abcam). All secondary antibodies used for IF analysis were Alexa Fluor 488-, 594-, or 647-conjugated antibodies (Life Technologies).

For Western blot analysis, mouse anti-HPV16 E2, mouse anti-HA (HA.11 clone 16B12; Covance), rabbit anti-HA, goat anti-Rint1, rabbit anti-ERp72 (catalogue number 5033; Cell Signaling), mouse anti-Grb2 (catalogue number SC-8034; Santa Cruz), rabbit anti-histone H3 (catalogue number A300-823A; Bethyl), rabbit anti-ZW10, mouse β-actin (clone AC-15, catalogue number A5441; Sigma-Aldrich), and mouse anti-FLAGM2 (catalogue number F1804; Sigma-Aldrich) primary antibodies were used, which were detected with horseradish peroxidase (HRP)-conjugated secondary antibodies (Thermo Scientific).

For co-IP, sheep anti-HPV16 E2 (52), mouse anti-HA, mouse anti-FLAGM2, rabbit anti-HA, and goat anti-Rint1 were used. Sheep preimmune serum (Dundee Cell Products), normal rabbit IgG (catalogue number SC-2027; Santa Cruz Biotechnology), mouse anti-HA, and normal mouse IgG (catalogue number SC-2025; Santa Cruz Biotechnology) were used as negative-control antibodies.

### Coimmunoprecipitation.

C33a cells (3 × 10^6^) were seeded into 100-mm-diameter tissue culture dishes and transfected by using X-tremeGENE HP (Roche) at a DNA-to-reagent ratio of 1:2. At 24 h posttransfection, the cells were lysed, and coimmunoprecipitations performed as previously described (36).

### Immunofluorescence.

C33a cells were plated onto coverslips at 3 × 10^6^ cells/100-mm dish and transfected by using the Lipofectamine 2000 reagent (Life Technologies) at a DNA-to-reagent ratio of 1:3. At 24 h posttransfection, cells were fixed with −20°C methanol for 4 min at −20°C and washed three times with PBS. Cells were permeabilized with 0.2% Triton X-100–PBS for 15 min at 4°C and washed three times with PBS. Samples were blocked with 3% bovine serum albumin (BSA)–PBS for 1 h at room temperature and washed twice with PBS before incubation with a primary antibody diluted in block solution overnight at 4°C in a humidified chamber. For Rint1 antibody peptide blocking experiments, 0.2 μg primary antibody was incubated with 2 μg blocking peptide (catalogue number SC-19404P; Santa Cruz) in a total volume of 100 μl PBS for 1 h at room temperature (RT) before further dilution in blocking buffer for immunofluorescence analysis. The coverslips were washed with PBS and incubated with secondary antibody diluted in block solution for 1 h at RT. Cellular DNA was stained with 10 μg/ml Hoechst 33342 (Life Technologies) in PBS for 30 min at room temperature. The coverslips were mounted by using the ProLong Gold antifade reagent (Life Technologies). Images were acquired by using a Nikon Eclipse E600 confocal laser scanning microscope and the Leica DC200 integrated digital camera and software. For quantification of colocalization, confocal images were acquired from three independent experiments and analyzed by using ImageJ (v.1.50c). Manders' overlap coefficient was used to describe the fraction of E2 that colocalized with endogenous Rint1 for each image, and data were used to calculate the mean percent overlap.

### Subcellular fractionation.

Subconfluent C33a cells in 100-mm dishes were transfected with an HPV16 E2-expressing plasmid or mock transfected. Cells were harvested 24 h following transfection, and subcellular fractions were isolated by using a subcellular protein fractionation kit (Thermo Scientific) according to the manufacturer's instructions. Equal amounts of each fraction were separated by SDS-PAGE, and proteins were detected by Western blotting.

### Protein transport assays.

U2OS cells stably expressing the HPV16 E2 protein or an empty vector control (40) were grown on coverslips. Cells were transfected with pEGFP-VSVG by using X-tremeGENE HP and incubated at 40°C for 24 h. Cells were then transferred to 32°C to stimulate vesicle transport, and individual coverslips were fixed in 3.7% paraformaldehyde (PFA) in PBS at 0, 30, and 60 min. Microscopic analysis was performed with a Nikon E600 epifluorescence microscope, and images were captured by using a Nikon DXM1200F digital camera.

### DNA replication assay.

Transient DNA replication assays were performed as described previously (52). In brief, 2.5 × 10^5^ C33a cells were seeded into each well of a 6-well plate and transfected in triplicate with 50 ng p16OriM, 500 ng pHPV16-HAE1, and 10 ng pCMV-E2 in the absence or presence of 100, 250, and 500 ng pcDNA-HA-Rint1. For each experiment, one well was harvested for protein expression analysis by Western blotting using urea lysis buffer (8 M urea, 50 mM Tris-HCl [pH 7.5], and 14 mM β-mercaptoethanol) and sonication. DNA was extracted from the remaining duplicate wells by Hirt extraction. Purified DNA was digested with DpnI. The amount of replicated pOri16M was quantified by qPCR using Sensimix Sybr (Bioline) and an MXPro3005 PCR machine (Agilent) as previously described (50, 52).

### Statistical analysis.

Where indicated, data were analyzed by using two-tailed, unpaired Student's *t* test.
